# The Sensitivity and Specificity of Multiparametric Magnetic Resonance Imaging and Prostate-Specific Membrane Antigen Positron Emission Tomography/Computed Tomography for Predicting Seminal Vesicle Invasion in Clinically Significant Prostate Cancer: A Multicenter Retrospective Study

**DOI:** 10.3390/jcm13154424

**Published:** 2024-07-29

**Authors:** Darshan Sitharthan, Song Kang, Patrick-Julien Treacy, Jacob Bird, Kate Alexander, Sascha Karunaratne, Scott Leslie, Lewis Chan, Daniel Steffens, Ruban Thanigasalam

**Affiliations:** 1 Surgical Outcomes Research Centre (SOuRCe), Royal Prince Alfred Hospital, Missenden Road, Sydney, NSW 2050, Australia; 2 RPA Institute of Academic Surgery (IAS), Royal Prince Alfred Hospital, Sydney, NSW 2050, Australia; 3 Department of Urology, Royal Prince Alfred Hospital (RPAH), Sydney, NSW 2050, Australia; 4 Faculty of Medicine and Health, Central Clinical School, The University of Sydney, Sydney, NSW 2050, Australia; 5 Department of Urology, Concord Repatriation General Hospital (CRGH), Sydney, NSW 2139, Australia

**Keywords:** prostate cancer, robotic prostatectomy, seminal vesicle invasion, magnetic resonance imaging, prostate-specific membrane antigen positron emission tomography/computed tomography, extracapsular extension, local staging, predictive power, histopathology

## Abstract

**Background/Objectives:** The presence of seminal vesicle invasion (SVI) in prostate cancer (PCa) is associated with poorer postoperative outcomes. This study evaluates the predictive value of magnetic resonance imaging (MRI) and prostate-specific membrane antigen positron emission tomography/computed tomography (PSMA PET/CT) for SVI in PCa. **Methods:** This cohort study included consecutive robotic prostatectomy patients for PCa at three Australian tertiary referral centres between April 2016 and September 2022. MRI and PSMA PET/CT results, clinicopathological variables, including age, BMI, prostate-specific antigen (PSA), PSA density, DRE, Biopsy Gleason score, Positive biopsy cores, PIRADS v2.1 score, MRI volume and MRI lesion size were extracted. The sensitivity, specificity, and accuracy of MRI and PSMA PET/CT for predicting SVI were compared with the histopathological results by receiver operating characteristic (ROC) analysis. Subgroup univariate and multivariate analysis was performed. **Results:** Of the 528 patients identified, 86 had SVI on final pathology. MRI had a low sensitivity of 0.162 (95% CI: 0.088–0.261) and a high specificity of 0.963 (95% CI: 0.940–0.979). The PSMA PET/CT had a low sensitivity of 0.439 (95% CI: 0.294–0591) and a high specificity of 0.933 (95% CI: 0.849–0.969). When MRI and PSMA PET/CT were used in combination, the sensitivity and specificity improved to 0.514 (95%CI: 0.356–0.670) and 0.880 (95% CI: 0.813–0.931). The multivariate regression showed a higher biopsy Gleason score (*p* = 0.033), higher PSA (*p* < 0.001), older age (*p* = 0.001), and right base lesions (*p* = 0.003) to be predictors of SVI. **Conclusions:** MRI and PSMA PET/CT independently underpredicted SVI. The sensitivity and AUC improved when they were used in combination. Multiple clinicopathological factors were associated with SVI on multivariate regression and predictive models incorporating this information may improve oncological outcomes.

## 1. Introduction

Prostate cancer (PCa) is the second most commonly diagnosed cancer in men worldwide [1]. The staging of PCa has a significant role in determining prognosis and five-year survival. Seminal vesicle invasion refers to the invasion of PCa into the muscular wall of extraprostatic seminal vesicles [2]. The presence of seminal vesicle invasion (SVI) and extracapsular extension (ECE) indicates a high risk of PCa and is associated with adverse patient outcomes including biochemical recurrence and the likelihood of metastases [3]. The prevalence of SVI varies significantly in the literature with a meta-analysis involving 34 studies showing a prevalence of SVI between 1 and 27% [4]. The early prediction of SVI pre-operatively is important to allow for adjusted treatment planning including the better use of adjuvant therapies to prevent this and ensure improved patient outcomes. 

MRI played a role in prostate cancer detection, staging, and treatment planning since 1982 [5]. Current standard practice involves the use of 1.5 T or 3 T mpMRI which includes diffusion weight imaging, dynamic contrast enhanced imaging, and T2-weighted MRI [6]. Standardised and validated reporting methods such as the Prostate Imaging Reporting and Data System Version 2.1 (PIRADS v2.1) have been implemented for the objective assessment of prostatic lesions on MRI [7]. Despite this, the literature shows a significant degree of heterogeneity associated with MRI interpretation, including for the prediction of SVI [5,8]. 

A recent meta-analysis on the ability of mpMRI to predict SVI showed a high specificity of 0.96 (95% CI 0.95–0.97) and a lower sensitivity of 0.58 (95% CI 0.47–0.68) [4]. In comparison, prostate-specific antigen (PSA) and digital rectal exam (DRE) were commonly used prior to and in conjunction with MRI in predicting SVI [9]. The use of DRE for the detection of SVI relies on the palpation of nearby structures which can be difficult to detect without ample experience [10,11]. Additionally, PSA and DRE do not allow for enough information for the staging and treatment planning of more severe disease [12].

Previously used to detect metastasis or recurrence in suspected advanced disease, evidence is emerging on the use of prostate-specific membrane antigen positron emission tomography/computed tomography (PSMA PET/CT) for PCa staging [13]. Studies comparing PSMA PET/CT with MRI showed similar results in overall PCa detection or minor improvements with PSMA PET/CT but similar results in terms of SVI detection [14,15,16,17]. A systematic review summarised the similarities between mpMRI and PET/CT staging, both facing variability based on interpretation and protocol and recommending the potential combined use due to the additional utility of PET/CT in lymph node and metastasis detection [18].

This study primarily aimed to investigate the role of mpMRI and PSMA PET/CT in PCa staging by determining their sensitivity, specificity, and accuracy for predicting SVI in PCa. As a secondary objective, this study aimed to determine the clinicopathological variables that are associated with SVI. 

## 2. Methods

### 2.1. Study Design

The study involved the retrospective analysis of all consecutive patients who underwent RARP for PCa at multiple expert centres in Sydney, Australia, from April 2016 to May 2023. All the patients undergoing elective RARP for PCa under the supervision of three fellowship trained urological surgeons, with more than 50 robotic prostatectomies each, were recruited from three tertiary referral centres. Patients who did not undergo a complete RARP including due to intraoperative complications, had co-morbidities affecting imaging or histopathology results or had previous therapy for prostate cancer resulting in altered histopathology results were excluded. Ethics approval was granted by the Sydney Local Health District Human Ethics Committee (X23-0297 & 2023/ETH01706). This study followed the Strengthening of Reporting Observational Studies in Epidemiology (STROBE) guidelines [19].

### 2.2. Outcome Measures

Patient demographics (age and body mass index (BMI)), clinical characteristics (PSA, DRE, and predicted stage) pathological outcomes including TRUS or TPUS results (Gleason score and number of positive cores on biopsy) were extracted from medical records. PSA density was calculated by dividing the PSA level by the prostate volume, which was determined through mpMRI. All biopsy results were reviewed by at least one fellowship trained pathologist. Biopsy and histopathology Gleason scores were reported by standardised International Society of Urological Pathology (ISUP) grade groups [20]. The ISUP grade groups and corresponding Gleason scores are listed in Appendix A. 

### 2.3. Multiparametric Magnetic Resonance Imaging 

Preoperative mpMRI information for the participants of the study was obtained from radiologist reports in patient electronic medical records. Patients underwent either 1.5 T or 3 T MRI with an endorectal coil. Fellowship-trained radiologists reported on mpMRI results. mpMRI sequences included T2 weighted, dynamic contrast-enhanced, and diffusion weight imaging. Results were scored and reported based on standardised PIRADS v2.1 protocol, with ECE and SVI reported separately [7].

### 2.4. Prostate-Specific Membrane Antigen Positron Emission Tomography/Computed Tomography 

Preoperative PSMA PET/CT information for the participants of the study was obtained from the radiologist reports in patient electronic medical records. Patients underwent ^68^Gadolinium PSMA PET/CT within 60 min of injection. Fellowship-trained radiologists reported on PSMA PET/CT results. The location and SUVmax were reported in addition to presence of SVI. 

### 2.5. Robotic Prostatectomy and Histopathology

The Da Vinci Xi from Intuitive Surgery was used to perform the RARP. Relevant specimens collected during surgery were sent to each respective hospital’s pathology department for histopathological analysis. The specimens were then sectioned according to the whole-mount staining technique. Histopathological analysis for each specimen was then performed by an expert uropathologist. Gleason score was reported based on standardised ISUP grade groups. The presence of SVI and ECE were evaluated separately and detailed in each histopathology report. Additionally, the reports included information on surgical margins, positive lymph nodes, and overall disease staging. 

### 2.6. Data Analysis

Statistical Analysis was performed using the SPSS V29 (SPSS Inc., Chicago, IL, USA). Patient characteristics were presented using descriptive statistics, where the mean ± standard deviation was used to represent continuous variables. Sensitivity, specificity, positive predictive value (PPV), and negative predictive value (NPV) for the prediction of SVI and ECE were calculated for mpMRI and PSMA PET/CT separately and in combination using histopathology as a reference standard. The receiver operating characteristic curve analysis (ROC) was used to estimate accuracy based on the area under the curve (AUC) for mpMRI and PSMA PET/CT separately and in combination. Results with a *p* value < 0.2 on the univariate regression were considered for progression to multivariate analysis. Results with a *p* value < 0.05 were considered statistically significant.

## 3. Results

A total of 539 patients underwent RARP for PCa during the study period; of these, 11 patients who did not completely undergo RARP or have previous alternative therapy for prostate cancer resulting in altered histopathology results were excluded—leaving a cohort of 528 patients. From this cohort, 288 (55%) underwent only mpMRI with no PSMA PET/CT, 10 (2%) underwent only PSMA PET/CT with no mpMRI and 151 (29%) underwent both mpMRI and PSMA PET/CT. Of the total cohort, 86 (16%) had SVI and 296 (56%) had ECE on histopathology. Overall, patients had a mean age of 65.35 years (SD = 8.47) and mean a PSA of 8.09 (SD = 6.70). Participant total and grouped baseline clinicopathological characteristics are summarised and presented in Table 1. 

### 3.1. Diagnostic Performance of mpMRI and PSMA PET/CT

Of the 439 participants who underwent preoperative mpMRI, 25 were found to have predicted SVI on mpMRI compared to 68 who were diagnosed with SVI on histopathology. Sensitivity, specificity, PPV, NPV and accuracy described by AUC are included in Table 2. mpMRI showed a low sensitivity and PPV as well as high specificity and NPV for predicting SVI. ROC analysis showed mpMRI to have an AUC for predicting the SVI of 0.562 (95% CI: 0.493–0.641). However, this AUC was not statistically significant (*p* = 0.103). mpMRI showed a similarly low sensitivity and high specificity for predicting ECE. This corresponded to a similar AUC compared to that for predicting the SVI of 0.622 (95% CI: 0.570–0.674; *p* ≤ 0.001) for predicting ECE. These results for mpMRI predicting ECE in PCa are presented in Table 3. The PSMA PET/CT prediction of ECE was not reported for the majority of patients.

**Figure 1:**
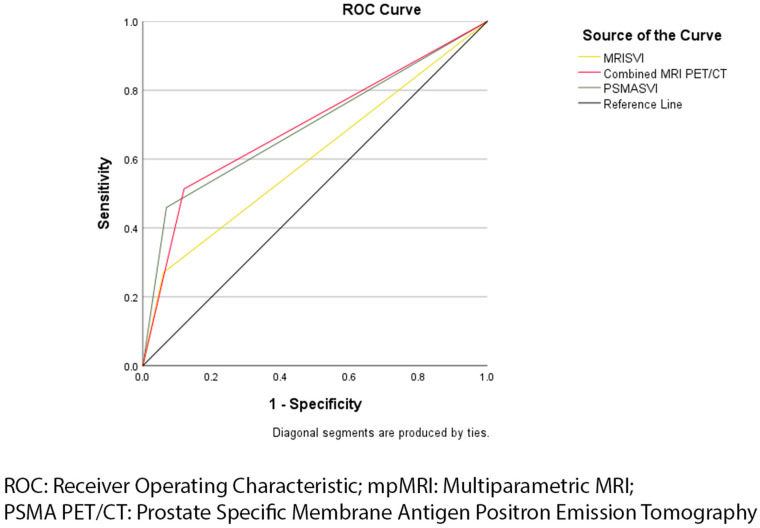
Receiver operating characteristic curve of multiparametric MRI and PSMA PET/CT in predicting seminal vesicle invasion.

Of the 161 participants who underwent PSMA PET/CT, 26 were predicted to have SVI on PSMA PET/CT compared to 41 who were diagnosed with SVI on histopathology. Sensitivity, specificity, PPV, NPV, and AUC are included in Table 2. PSMA PET/CT showed low sensitivity and PPV as well as high specificity and NPV for predicting SVI. PSMA PET/CT had a similar AUC of 0.682 (95% CI: 0.578–0.786; *p* ≤ 0.001) in comparison to mpMRI. The comparison of ROC curves between mpMRI and PSMA PET/CT for predicting SVI are presented in Figure 1.

When used in combination, mpMRI and PSMA PET/CT improved in sensitivity, NPV, and overall accuracy with an AUC of 0.679 (95% CI: 0.590–0.804; *p* ≤ 0.001). However, specificity was lower than the specificity of mpMRI and PSMA PET/CT alone for predicting SVI. The use of PSMA PET/CT and mpMRI in combination showed a higher PPV in comparison to mpMRI alone, but lower than PSMA PET/CT alone. These results and comparisons are presented in Table 2 and Figure 1. 

### 3.2. Univariate and Multivariate Analysis of Clinicopathological Factors

The association between SVI and clinicopathological factors are presented in Table 4. SVI had significant univariate associations with higher surgical age (*p* = 0.001), higher BMI (*p* = 0.172), higher PSA (*p* < 0.001), higher PSA density (*p* = 0.018), higher biopsy Gleason score (*p* = 0.060), left base lesions on MRI (*p* = 0.005), left anterior lesions on MRI (*p* = 0.079), right base lesions on MRI (*p* = 0.003), and right anterior lesions on MRI (*p* = 0.115). In the multivariate analysis, a higher surgical age (*p* = 0.001), higher PSA (*p* < 0.001), higher biopsy Gleason score (*p* = 0.033) and right base lesions on MRI (*p* = 0.003) were significantly associated with SVI. 

## 4. Discussion

### 4.1. Summary of Findings

The results of this study revealed that both mpMRI and PSMA PET/CT have high specificity but low sensitivity for the prediction of SVI. These results are comparable to the current literature in the tendency to underpredict SVI [4,21]. Results for the mpMRI prediction of ECE showed a similarly low sensitivity and high specificity, reflecting a similar tendency to underpredict ECE [4]. Sensitivity, specificity, and AUC improved when MRI and PSMA PET/CT were used in combination compared to individually.

The meta-analysis has shown a pooled specificity of 0.91, which is similar to the specificity of 0.963 shown in this study for the ability of mpMRI to predict SVI [4]. Meta-analysis also showed a pooled specificity of 0.94 for the PSMA PET/CT prediction of SVI, which reflected the 0.933 shown by this study [22]. Compared to previous studies comparing mpMRI and PSMA PET/CT, the two imaging methods showed similar results, with PSMA PET/CT having slightly increased sensitivity relative to mpMRI [15,17].

While PSMA PET/CT has shown promising potential in the diagnostic setting and local staging of prostate cancer, its sensitivity for predicting SVI remains limited. Our findings suggest that PSMA PET/CT should not replace mpMRI but rather be used in conjunction with it to leverage the strength of both modalities. The combined use of mpMRI and PSMA PET/CT improved the sensitivity and overall accuracy for predicting SVI, highlighting the benefit of a multimodal imaging.

The main result that differed from hypothesis was the exceptionally low sensitivity of mpMRI for predicting SVI in comparison to the previous literature. A meta-analysis by De Rooij et al. [4] showed a pooled sensitivity of 0.57 compared to 0.162 in this study. Despite this, it was typically found that studies of larger cohorts tended to report a lower sensitivity with large cohort studies by Lee C. et al. [23], Lee Z. et al. [24], and Jeong et al. [25] reporting sensitivity between 0.275 and 0.401, while smaller cohort studies by Caldas et al. [26], Bates et al. [27] and Celen et al. [14] reported sensitivity between 0.833 and 1.0. Additionally, this study is unique in the method of data collection for mpMRI and PSMA PET/CT which included heterogeneity that may be typically present in regular clinical practice. These variables may have contributed to the unexpectedly low sensitivity of mpMRI and suggest that the result is an accurate reflection of the clinical value of mpMRI and PSMA PET/CT [28].

This study additionally found several variables to be significant independent predictors of SVI on multivariate analysis. Most variables found to be predictors were as hypothesised, including higher biopsy Gleason score, higher surgical age, increased PSA, and base lesions on MRI [29,30,31]. However, there were also some unexpected results including the lack of statistically significant association between DRE with SVI [32,33].

PSA was shown to have a statistically significant association with SVI on multivariate regression. Literature supports the role that PSA plays in PCa detection and monitoring, frequently used throughout the patient journey [34,35,36]. Additionally, these studies have stated that PSA tends to overpredict SVI, which supports their clinical use, particularly in combination with MRI and PSMA PET/CT, which this study has shown to underpredict SVI.

Similarly, results from biopsy were strongly associated with SVI, with a higher biopsy Gleason score being associated with SVI on multivariate regression. This supports the widely acknowledged use of TRUS- or TPUS-guided biopsy in PCa diagnosis and staging, despite its risks as an invasive procedure including sepsis-related mortality [37]. Despite this, ultrasound-guided biopsy also has its own limitations, including an inability to provide sufficient anatomical and staging information for treatment planning, which can be addressed through use in combination with other SVI predictors [38].

Considering these advancements, we recognise the potential of PSMA tracers as intra-operative guides for locating PCa, which could significantly improve the surgical precision and outcomes. Although our study focuses on the use of PSMA PET/CT pre-operatively, future research should explore the use of PSMA tracers for intra-operative tumour identification. Additionally, indocyanine green (ICG) and superparamagnetic iron oxide nanoparticles (SPION) offer promising alternatives for intra-operative imaging and may be valuable when PSMA tracers are unavailable or to complement traditional modalities such as mpMRI and PSMA PET/CT [39,40]. These approaches could enhance the accuracy of staging and treatment planning in prostate cancer, contributing to better patient outcomes.

### 4.2. Strengths and Weaknesses

To our knowledge, this is one of the biggest cohorts with an entirely robotic prostatectomy cohort, including 528 patients undergoing RARP for PCa. Given that the oncological and functional outcomes associated with RARP have improved, it is now often the preferred method of operation, and this study may therefore be more reflective of modern practices [41]. Additionally, the larger sample size suggests a more representative sample, improving the significance of results. This is reflected in the prevalence of SVI within our study cohort being 16%, which matches the average global prevalence shown in the current literature despite high variation [4,42].

Another strength of this study is that it uniquely covers both widely used preoperative imaging methods for predicting SVI whereas existing literature largely compares only mpMRI before the introduction of standardised PSMA PET/CT reporting in 2020 [25,43,44,45]. This presents a more modern and updated view of novel imaging predictors for SVI in PCa. Similarly, this study tests 11 different variables on univariate analysis based on literature and expert opinion. This provides a comprehensive picture of SVI predictors for guiding management and areas of research.

However, there are several limitations to consider. The method of collecting patient data used in this study introduces limitations, including a lack of complete data as not all patients may have been subject to the same degree of preoperative testing. This also applied to the inability to compare MRI and PSMA PET/CT results for ECE as results were not routinely reported in over 50% of PSMA PET/CT reports. To reduce this bias, an attempt to retrieve any missing patient data was made through the review of private electronic medical records with waived consent. During statistical analysis, participants with missing variables were excluded to further prevent the risk of information bias. This lack of complete data also introduces the possibility of selection bias. Tests were ordered based on surgeons’ clinical judgement, which may introduce bias as patients who were more likely to have advanced disease were ordered additional investigations. Certain external factors also contributed to this bias, such as the introduction of patient financial assistance for MRI in 2018 and PSMA PET/CT in 2022. To minimise these risks, investigations were ordered by the surgeon based on relevant clinical guidelines at the time of patient management.

These limitations may have contributed to the lack of statistical significance for the results of ROC analysis regarding MRI and the PSMA PET/CT prediction of SVI. A likely contributor is the low number of patients who had SVI predicted on MRI. This may be related to the fact that mpMRI and PSMA PET/CT results were collected from radiologist reports in patient records as opposed to one or more standardised readers which were used in other similar studies [23,25,44,45]. Although all readers were fellowship-trained radiologists, the remaining possibility of heterogeneity reflects that which is common in clinical practice [46]. Additionally, the creation of validated systems such as PIRADS v2.1, which was used for all patient MRIs, aims to reduce the heterogeneity of interpretation between readers [7].

Furthermore, the study did not account for the influence of prostatic carcinoma histological variants and aberrant growth patterns, such as ductal, intraductal, and cribriform patterns, which are known to impact oncological outcomes. These variants have been associated with worse pathological features and poorer prognostic outcomes compared to the conventional acinar adenocarcinoma of the prostate [47]. Future research should consider these histological subtypes to provide a more comprehensive assessment of SVI prediction and overall disease prognosis.

### 4.3. Implications of Study

mpMRI and PSMA PET/CT play a well-researched and established role in the non-invasive diagnosis and mapping of PCa, including the detection of perineural invasion, lymph nodes invasion, and metastases [48,49]. As such, mpMRI and PSMA PET/CT should continue to play a key role in the clinical detection and staging of PCa; however, based on the results of this study, neither should not be relied upon as the sole predictor of SVI. Instead, mpMRI and PSMA PET/CT results should be acknowledged in combination with other predictors.

Based on these results and the presence of statistically significant predictors of SVI on multivariate regression, research into the creation of nomograms or other predictive models may be useful as an area of research. Studies have been conducted into the use of nomograms for predicting SVI [50,51]. However, these studies consist of small cohorts which may lead to unreliable results and do not incorporate PSMA PET/CT which this study has shown to be beneficial when used with mpMRI.

When used in combination, mpMRI and PSMA PET/CT showed a strong specificity and accuracy for predicting SVI. As such, there is good potential for the use of both imaging modalities in a combined preoperative imaging approach. This includes the use of mpMRI and PSMA PET/CT in image-guided surgery [52,53]. With research being performed into machine learning and whether this can improve surgical outcomes, a potential question could be whether the accuracy mpMRI and PSMA PET/CT have shown is sufficient for use in such AI-based surgical models.

## 5. Conclusions

This study suggests mpMRI and PSMA PET/CT are highly specific but not sensitive predictors of SVI in PCa. However, this study also demonstrated an improved sensitivity and overall predictive accuracy when the two imaging modalities are used in combination to predict SVI suggesting that a combined approach with other clinical variables included in a global predictive tool may be beneficial to the patients.

## Figures and Tables

**Table 1:** Participant baseline characteristics by imaging subset.

Variable		Total (528)	Multiparametric MRI (439)	PSMA PET/CT (161)	Combined MRI and PSMA PET/CT (151)
Age, y, mean (±SD)		65.35 ± 8.47	64.78 ± 8.73	65.80 ± 7.85	65.52 ± 7.90
BMI, kg/m^2^, mean (±SD)		27.25 ± 4.94	27.21 ± 4.72	27.22 ± 4.74	27.07 ± 3.87
PSA, ng/mL, mean (±SD)		8.09 ± 6.70	7.97 ± 7.18	9.24 ± 8.48	9.06 ± 8.56
PSA density, ng/mL^2^, mean (±SD)		0.25 ± 0.28	0.25 ± 0.28	0.33 ± 0.43	0.32 ± 0.45
Biopsy ISUP grade group, n (%)					
	1	21 (4.04)	19 (4.38)	2 (1.26)	2 (1.34)
	2	242 (46.90)	201 (46.31)	41 (25.79)	40 (26.85)
	3	129 (25.00)	109 (25.12)	42 (26.42)	39 (26.17)
	4	46 (8.91)	34 (7.83)	19 (11.95)	19 (12.75)
	5	82 (15.89)	71 (16.36)	55 (34.59)	49 (32.89)
MRI PIRADS score, mean (±SD)		4.07 ± 0.83	4.07 ± 0.83	4.18 ± 0.84	4.18 ± 0.84
MRI SVI, n (%)					
	Yes	25 (5.69)	25 (5.69)	17 (11.26)	17 (11.26)
	No	414 (94.30)	414 (94.31)	134 (88.74)	134 (88.74)
MRI ECE, n (%)					
	Yes	125 (28.47)	125 (28.47)	55 (36.42)	55 (36.42)
	No	314 (71.53)	314 (71.53)	96 (63.58)	96 (63.58)
PSMA PET/CT SVI, n (%)					
	Yes	26 (16.15)	25 (16.56)	26 (16.15)	25 (16.56)
	No	135 (83.85)	126 (83.44)	135 (83.85)	126 (83.44)
Pathological ISUP grade group, n (%)					
	1	19 (3.60)	15 (3.42)	3 (1.86)	3 (1.99)
	2	263 (49.81)	216 (49.20)	52 (32.30)	50 (33.11)
	3	130 (24.62)	110 (25.06)	39 (24.22)	38 (25.17)
	4	24 (4.55)	19 (4.33)	15 (9.32)	14 (9.27)
	5	92 (17.42)	79 (18.00)	52 (32.30)	46 (30.46)
Pathological stage, n (%)					
	<T2c	231 (43.75)	193 (43.96)	57 (35.40)	54 (35.76)
	T3a	210 (39.77)	176 (40.09)	62 (38.51)	60 (39.74)
	T3b	87 (16.48)	70 (15.95)	42 (26.09	37 (24.50)
Histological ECE, n (%)					
	Yes	296 (56.06)	193 (43.96)	103 (63.98)	96 (63.58)
	No	232 (43.94)	246 (56.04)	58 (36.02)	55 (36.42)
Histological SVI, n (%)					
	Yes	86 (16.29)	70 (15.95)	42 (26.09)	37 (24.50)
	No	442 (83.71)	369 (84.05)	119 (73.91)	114 (75.50)

BMI: Body mass index; PSA: Prostate-specific antigen; ISUP: International Society of Urological Pathology; MRI: Magnetic resonance imaging; PIRADS: Prostate imaging reporting and data system; SVI: Seminal vesicle invasion; ECE: Extracapsular extension; PSMA PET/CT: Prostate-specific membrane antigen positron emission tomography.

**Table 2:** Sensitivity and specificity of multiparametric MRI and PSMA PET/CT in predicting seminal vesicle invasion.

SVI	Multiparametric MRI		PSMA PET/CT		Combined MRI and PSMA PET/CT	
	B (95% CI)	*p* Value	B (95% CI)	*p* Value	B (95% CI)	*p* Value
Sensitivity (%)	0.162 (0.088–0.261)	-	0.439 (0.294–0.591)	-	0.514 (0.356–0.670)	-
Specificity (%)	0.963 (0.940–0.979)	-	0.933 (0.849–0.969)	-	0.880 (0.813–0.931)	-
NPV (%)	0.863 (0.828–0.894)	-	0.830 (0.760–0.887)	-	0.851 (0.781–0.907)	-
PPV (%)	0.440 (0.258–0.633)	-	0.692 (0.503–0.846)	-	0.576 (0.406–0.734)	-
Accuracy, AUC (%)	0.562 (0.483–0.641)	0.103	0.682 (0.578–0.786)	<0.001	0.697 (0.590–0.804)	<0.001

SVI: Seminal vesicle invasion; MRI: Magnetic resonance imaging; PSMA PET/CT: Prostate-specific membrane antigen positron emission tomography; NPV: Negative predictive value; PPV: Positive predictive value; AUC: Area under the curve.

**Table 3:** Sensitivity and specificity of multiparametric MRI in extracapsular extension.

ECE	Multiparametric MRI	
	B (95% CI)	*p* Value
Sensitivity (%)	0.291 (0.217–0.374)	-
Specificity (%)	0.902 (0.865–0.931)	-
NPV (%)	0.759 (0.714–0.801)	-
PPV (%)	0.544 (0.426–0.659)	-
Accuracy, AUC (%)	0.622 (0.570–0.674)	<0.001

ECE: Extracapsular extension; MRI: Magnetic resonance imaging; NPV: Negative predictive value; PPV: Positive predictive value; AUC: Area under the curve.

**Table 4:** Univariate and Multivariate Association between patient demographics and seminal vesicle invasion.

Variable		Univariate Analysis		Multivariate Analysis	
		Odds Ratio (95% CI)	*p* Value	Odds Ratio (95% CI)	*p* Value
Age at surgery, y		1.059 (1.023–1.096)	0.001	1.072 (1.028–1.119)	0.001
BMI, Kg/m^2^		0.955 (0.894–1.020)	0.172	-	-
PSA, ng/mL		1.063 (1.028–1.099)	<0.001	1.069 (1.032–1.107)	<0.001
PSA density, ng/mL^2^		3.034 (1.210–7.611)	0.018	-	-
DRE		0.904 (0.682–1.199)	0.484	-	-
Biopsy Gleason score		1.010 (1.000–1.021)	0.060	1.008 (0.991–1.026)	0.033
Biopsy positive cores		0.991 (0.946–1.038)	0.667	-	-
PIRADS v2.1 score		1.073 (0.771–1.494)	0.677	-	-
PSMA PET/CT SUV max		1.010 (0.967–1.055)	0.644	-	-
MRI prostate volume, cc		0.882 (0.651–1.194)	0.415	-	-
MRI lesion size, mm		1.004 (0.965–1.045)	0.835	-	-
MRI lesion location					
	Left base	2.344 (1.294–4.245)	0.005	-	-
	Left mid	1.016 (0.594–1.738)	0.953	-	-
	Left apex	0.995 (0.560–1.771)	0.988	-	-
	Left anterior	0.274 (0.064–1.163)	0.079	-	-
	Left peripheral zone	1.353 (0.806–2.272)	0.252	-	-
	Left transitional zone	1.281 (0.611–2.689)	0.512	-	-
	Right base	2.411 (1.342–4.332)	0.003	2.720 (1.403–5.273)	0.003
	Right mid	1.231 (0.718–2.111)	0.449	-	-
	Right apex	0.942 (0.519–1.708)	0.843	-	-
	Right anterior	0.198 (0.026–1.485)	0.115	-	-
	Right peripheral zone	1.394 (0.830–2.340)	0.209	-	-
	Right transitional zone	1.134 (0.526–2.445)	0.749	-	-

## Data Availability

The data presented in this study are available on request from the corresponding author due to ethical reasons.

## Appendix A

**Table A1:** Table of definitions from International Society of Urological Pathology Consensus Conference on Gleason Grading of Prostatic Carcinoma.

ISUP Grade Group	Gleason Score
Grade Group 1	Gleason score ≤ 6
Grade Group 2	Gleason score 7 (3 + 4)
Grade Group 3	Gleason score 7 (4 + 3)
Grade Group 4	Gleason score 8
Grade Group 5	Gleason score 9–10

ISUP: International Society of Urological Pathology.
